# Isolation and Genomic Characterization of a Novel Porcine Reproductive and Respiratory Syndrome Virus 1 from Severely Diseased Piglets in China in 2024

**DOI:** 10.3390/vetsci12010061

**Published:** 2025-01-15

**Authors:** Shuai Yang, Meng Cui, Chen Li, Ming Qiu, Xiaoyang Zhu, Yanhan Lin, Yifan Meng, Yuejia Qiu, Wenhao Qi, Hong Lin, Wanglong Zheng, Jianzhong Zhu, Kewei Fan, Nanhua Chen

**Affiliations:** 1 College of Veterinary Medicine, Yangzhou University, Yangzhou 225009, China; 2 Longyan University and Fujian Provincial Key Laboratory for Prevention and Control of Animal Infectious Diseases and Biotechnology, Longyan 364012, China; 3 Jiangsu Co-Innovation Center for Prevention and Control of Important Animal Infectious Diseases and Zoonoses, Yangzhou University, Yangzhou 225009, China; 4 Comparative Medicine Research Institute, Yangzhou University, Yangzhou 225009, China

**Keywords:** porcine reproductive and respiratory syndrome virus 1, a novel isolate, complete genome sequencing, deletions, evolution

## Abstract

Since the first isolation of the porcine reproductive and respiratory syndrome virus 1 (PRRSV-1) BJEU06-1 strain from a Beijing pig farm in 2006, more and more PRRSV-1 isolates have been identified in China. In this study, we performed the routine detection of PRRSV-1 using 1521 clinical samples collected in 12 provinces/cities from February 2022 to May 2024. Only three lung samples from severely diseased piglets collected in January 2024 were detected as PRRSV-1-positive (0.197%, 3/1521). A PRRSV-1 strain (AHEU2024-2671) was successfully isolated in primary alveolar macrophages (PAMs) but not in Marc-145 cells. Genome sequencing showed that the AHEU2024-2671 isolate shared the highest genome similarity (90.67%) with the SC2020-1 isolate but only 84.01% similarity with the predominant BJEU06-1 strain. Noticeably, the AHEU2024-2671-like isolates not only contained deletions in nsp2 and the GP3-GP4 overlap region, but also contained a unique 6 nt deletion between nsp12 and the ORF2 gene. Furthermore, a genome-based phylogenetic tree supported that the AHEU2024-2671-like isolates form a novel subgroup within subtype 1. Overall, this study not only supported the idea that PRRSV-1 is rapidly evolving in Chinese swine herds, but also pulled the alarm that novel PRRSV-1 isolates with potentially increased pathogenicity might already exist in China, although they are still rarely detected among Chinese pigs.

## Simple Summary

Porcine reproductive and respiratory syndrome virus (PRRSV) causes huge economic loss to the global swine industry. In China, PRRSV-2 isolates are predominant, but PRRSV-1 isolates are also circulating in the swine herds. Since the first isolations of wild-type PRRSV-1 strains (BJEU06-1 and NMEU09-1), PRRSV-1 has been detected in the majority of regions in China. During the routine investigation of PRRSV-1 in China, we isolated a novel PRRSV-1 strain (AHEU2024-2671) from the lung of a severely diseased piglet from Fuyang city, Anhui province, in January 2024. The AHEU2024-2671 isolate could only replicate in primary alveolar macrophages (PAMs) and not in Marc-145 cells. Genome sequencing and multiple comparisons showed that AHEU2024-2671 is a novel isolate sharing the highest genome similarity (90.67%) with the SC2020-1 isolate. Noticeably, this study identified novel deletions in AHEU2024-2671-like isolates for the first time. Phylogenetic analysis showed that the AHEU2024-2671-like isolates form a new subgroup within subtype 1. Overall, this study provides new insights into the rapid evolution of PRRSV-1 in China.

## 1. Introduction

Porcine reproductive and respiratory syndrome (PRRS) has jeopardized the global swine industry for three decades, causing reproductive disorders in sows and respiratory syndromes in all ages of pigs [1]. PRRS has been estimated to cause USD 2.7 billion economic losses annually for the global swine industry [2]. The corresponding etiological agent, PRRS virus (PRRSV), is a single-stranded, positive-sense RNA virus belonging to the family of *Arteriviridae* in the order of *Nidovirales* [3]. The PRRSV genome is approximately 15 kb in length, containing a 5′ cap structure, 5′ untranslated region (UTR), at least 10 open reading frames (ORFs), 3′ UTR and a 3′ poly (A) tail. ORF1a and ORF1b encode 16 nonstructural proteins (nsp1α, nsp1β, nsp2N, nsp2TF, nsp2–nsp6, nsp7α, nsp7β and nsp8–nsp12), while ORF2–ORF7 encode 8 structural proteins, including minor envelope proteins (GP2a, E, GP3, GP4 and 5a), major envelope proteins (GP5 and M) and a nucleocapsid protein (N) [1]. Due to their high genetic diversity, PRRSV isolates have been divided into two species: *Betaarterivirus suid 1* (PRRSV-1) and *Betaarterivirus suid 2* (PRRSV-2) [4]. PRRSV-1 isolates can be clustered into four subtypes (1, 2, 3 and 4) [5], while PRRSV-2 isolates have been divided into 11 lineages and 21 sub-lineages [6]. PRRSV-1 strains were first isolated in Europe in 1991 [7], and PRRSV-2 strains were first isolated in North America in 1992 [8]. Even though PRRS eradication programs have been implemented in European and North American countries for years [9,10], both PRRSV-1 and PRRSV-2 isolates are still circulating in European, North American and Asian countries [7,8,11,12,13,14,15,16,17].

PRRSV has been prevalent in China for nearly 30 years [18]. Within this, PRRSV-2 isolates are predominant, including classical PRRSV-2, highly pathogenic PRRSV-2, NADC30-like PRRSV-2 and NADC34-like PRRSV-2 [18,19,20,21]. However, since we first isolated wild-type PRRSV-1 strains (BJEU06-1 and NMEU09-1) in mainland China [22], more and more PRRSV-1 strains have been identified in China [23,24,25,26,27,28,29]. For instance, a PRRSV-1 Amervac vaccine-like strain (GZ11-G1) was also isolated from Guizhou province in 2011 [29]. In 2018, a CReSA3-like EUGDHD2018 isolate (MK639926) was identified in Guangdong province, while a PR40-like 180900-5 (MK303390) strain was isolated in Henan province [30]. In 2020, the SC2020-1 strain was isolated from aborted piglets in Sichuan province [31]. Currently, PRRSV-1 has been prevalent in the majority of regions in China with BJEU06-1-like isolates serving as the predominant viruses in Chinese swine herds [32].

In this study, we performed routine detection of PRRSV-1 between February 2022 and May 2024. A total of 1521 clinical samples collected from 12 provinces/cities in China were studied. PRRSV-1 was detected in lung samples collected from severely diseased piglets from Anhui province in January 2024. The virus was isolated and put through complete genome sequencing. Multiple sequences were compared to determine the specific deletions in the genome. Genome-based phylogenetic analysis and recombination analysis were also executed to evaluate the characteristics of this PRRSV-1 isolate.

## 2. Materials and Methods

### 2.1. Clinical Sample Collection and Detection

During the routine detection of PRRSV-1 infection, a total of 1521 clinical samples (including lung, lymph node and serum) were submitted from 12 regions of China (Fuyang city in Anhui province; Zhumadian city in Henan province; Qingdao city in Shandong province; Jinchang city in Gansu province; Neijiang city in Sichuan province; Yangzhou city in Jiangsu province; Shuangyashan city in Heilongjiang province; Hangzhou city in Zhejiang province; Chaozhou city in Guangdong province; Xianyang city in Shanxi province; Beijing city; and Shihezi city in Xinjiang Uygur Autonomous Region) to the Animal Hospital at Yangzhou University from 22 February in 2022 to 20 May in 2024 (Table 1). A PRRSV-1-specific real-time RT-PCR assay was used for clinical detection [33]. For the PRRSV-1-positive samples, the infection statuses of other common porcine viruses were also determined as previously described [24,33,34,35], including PRRSV-2, classical swine fever virus (CSFV), porcine epidemic diarrhea virus (PEDV), transmissible gastroenteritis virus (TGEV), porcine deltacoronavirus (PDCoV), African swine fever virus (ASFV), pseudorabies virus (PRV) and porcine circoviruses (PCV2 and PCV3) (Table S1). In detail, total RNAs were extracted from serum samples or tissue sample homogenates using TRIpure reagent (Aidlab, Beijing, China). A PrimeScript 1st Strand cDNA Synthesis Kit (TaKaRa, Osaka, Japan) was used for first-strand cDNA synthesis. A PRRSV-1-specific probe, primer pairs and Premix Ex Taq (Probe qPCR, 2×) (TaKaRa, Osaka, Japan) were utilized for PRRSV-1 real-time PCR detection as we described previously [24].

### 2.2. Virus Isolation and Immunofluorescence Assay (IFA)

Both primary alveolar macrophages (PAMs) and Marc-145 cells were used for virus isolation and then put through IFA detection as previously described [36]. PAMs were prepared with lung lavage fluid of 6-week-old healthy piglets [37,38]. PAMs were cultured in RPMI-1640 medium (HyClone, Midvale, UT, USA) supplemented with 10% FBS (EallBio, Beijing, China), 100 U/mL penicillin and 100 μg/mL streptomycin (Solarbio, Beijing, China). Marc-145 cells were cultured in DMEM (HyClone, Midvale, UT, USA) supplemented with 10% FBS [39]. The PRRSV-1-positive samples were used for infection. The cytopathic effects (CPEs) in infected Marc-145 cells were monitored daily up to 7 days. The infected PAMs and Marc-145 cells were collected at 48 hpi and put through IFA detection. In detail, the infected cells were washed with PBS and then fixed with 4% paraformaldehyde. Cell samples were permeabilized with 0.5% TritonX-100 for 10 min and blocked with 1% BSA for 2 h. PRRSV N protein-specific murine mAb 15A1 (1:500) (kindly provided by Professor Kegong Tian from the National Research Center for Veterinary Medicine) and Dylight 594 goat anti-mouse IgG (1:1000, Invitrogen, Carlsbad, CA, USA) were used as the primary and secondary antibodies [24]. The 4′, 6-diamidino-2-phenylindole (DAPI) was used for cellular nuclei counterstaining. The results were visualized with an IX53 inverted fluorescence microscope (Olympus, Tokyo, Japan).

### 2.3. Western Blotting (WB)

WB was performed as we described previously [40]. Briefly, cell samples were first lysed in radio-immunoprecipitation assay (RIPA) buffer (50 mM Tris pH 7.2, 150 mM NaCl, 1% sodium deoxycholate, 1% Triton X-100). Subsequently, the extracted proteins were separated by 15% SDS-PAGE gels and then transferred to polyvinylidene fluoride (PVDF) membranes (Merck Millipore, Billerica, MA, USA). Furthermore, membranes were blocked for 1 h using 5% non-fat milk. Moreover, the membrane was incubated with the primary mAbs (1:1000 anti-GADPH and 1:1000 PRRSV-N mAbs 15A1) at 4 °C overnight. Later on, the membrane was nurtured with HRP-conjugated goat anti-mouse IgG (1:10,000, BBI, Beijing, China) at 37 °C for 1 h. Upon addition of enhanced chemiluminescence (ECL) substrate (Biosharp, Anhui, China), the protein signals were observed and captured by the WB imaging system (Tanon, Shanghai, China).

### 2.4. Genomic Sequencing

To determine the complete genome of PRRSV-1 isolate, total RNA was extracted from PRRSV-1 infected PAMs using TRIpure Reagent (Aidlab, Beijing, China). First-strand cDNA was composited using the viral RNA and a PrimeScript 1st Strand cDNA Synthesis Kit (TaKaRa, Osaka, Japan). Complete PRRSV-1 genomes were amplified using 12 primer pairs (Table 2) modified from previous studies [22,24,41]. The 12 PCR products overlapped with each other spanning the entire genome. The obtained amplicons were put through Sanger Sequencing by GENEWIZ Company (Suzhou, China). The obtained PRRSV-1 sequences were pieced together using DNAMAN 6.0 software [42].

### 2.5. Multiple Alignment, Phylogenetic Analysis and Recombination Detection

To compare the similarities among our PRRSV-1 isolate and previously identified PRRSV-1 strains, complete genome and fragment alignments were carried out using DNAMAN 6.0 [9]. To determine the evolutionary relationships between our PRRSV-1 isolate and representative PRRSV strains, the complete genomes from our PRRSV-1 isolate, 49 representative PRRSV-1 isolates and 3 representative PRRSV-2 isolates were put through multiple sequence alignment by Clustal X [10]. A genome-based phylogenetic tree was constructed by MEGA 6.06 [9,15]. To analyze the role of recombination events in the generation of our PRRSV-1 isolate, RDP4 and SimPlot 3.5.1 were used to screen potential cross-over events in the aligned PRRSV genomes [16,43].

### 2.6. Statistical Analysis

The in vitro virus replication in PAMs was shown in means ± standard deviations (SD). The differences among groups were detected using a Mann–Whitney U test embedded in the Graphpad Prism 8 XML project [44]. A *p* value < 0.05 indicated statistical significance.

## 3. Results

### 3.1. PRRSV-1 Detection

**Figure 1:**
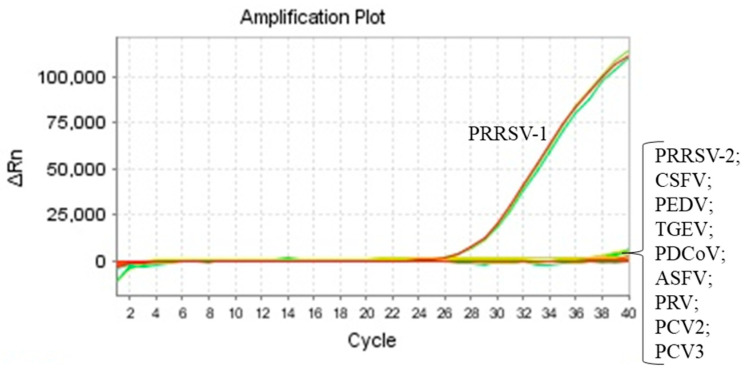
The detection of PRRSV-1 in a lung sample from severely diseased piglets. Real-time RT-PCR assays were utilized to detect PRRSV1, PRRSV-2, CSFV, PEDV, TGEV, PDCoV, ASFV, PRV, PCV2 and PCV3 as previously described [24,33,34,35]. Only PRRSV-1 was detected in the representative lung sample.

During the routine detection of PRRSV-1 in 1521 clinical samples, the PRRSV-1-positive rate was only 0.197% (3/1521) (Table 1). PRRSV-1 was only identified in three lung samples collected from severely diseased piglets in Fuyang city, Anhui province, on 19 January 2024 (Figure 1). Meanwhile, the other common porcine viruses (PRRSV-2, CSFV, PEDV, TGEV, PDCoV, ASFV, PRV, PCV2 and PCV3) turned out to be all negative in the three lung samples (Figure 1). These results indicated that severe clinical diseases in piglets might be associated PRRSV-1 infection.

### 3.2. PRRSV-1 Isolation

**Figure 2:**
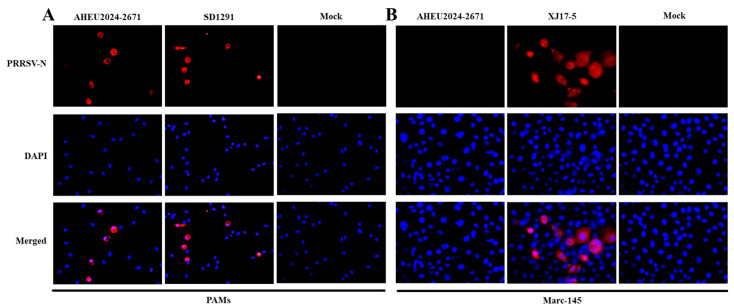
PRRSV-1 isolation in PAMs and Marc-145 cells. (**A**) The infected PAMs were examined by IFA at 48 hpi. PRRSV-1-specific antigen was detected in AHEU2024-2671-infected PAMs. PRRSV-1 SD1291 isolate-infected cells were used as positive control [24], while mock-infected cells were set as negative control. (**B**) The infected Marc-145 cells were also examined by IFA at 48 hpi. PRRSV-1 specific antigen could not be detected in AHEU2024-2671-infected Marc-145 cells. PRRSV-2 XJ17-5 isolate-infected cells were used as positive control [9], while mock-infected cells were set as negative control.

**Figure 3:**
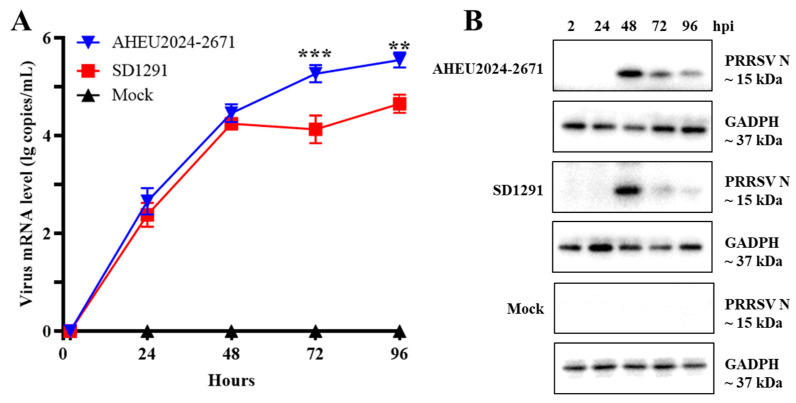
Replication efficacies of PRRSV-1 isolates in PAMs. (**A**) Dynamics of virus replication in PAMs were detected by PRRSV-1-specific real-time RT-PCR assay [45]. Multiple-step growth curves showed that the AHEU2024-2671 isolate has significantly higher in vitro replication efficacy than the SD1291 isolate from 72 to 96 hpi (*p* < 0.05). (**B**) WB results also showed that PRRSV-1 N protein expression levels were higher in the AHEU2024-2671-infected PAMs than in the SD1291-infected PAMs at 72–96 hpi. ** indicates *p* < 0.01, *** indicates *p* < 0.001.

To characterize the new PRRSV-1 strain, PRRSV-1-positive lung homogenates were put through virus isolation in PAMs and Marc-145 cells. IFA was performed using inoculated PAMs and Marc-145 cells at 48 hpi. As shown in Figure 2A, the new PRRSV-1 could be isolated in PAMs, which was denominated as AHEU2024-2671 strain. However, it could not be isolated in Marc-145 cells (Figure 2B). The replication efficacies of AHEU2024-2671 and SD1291 isolates in PAMs were also determined. Both growth curves and WB results supported that the AHEU2024-2671 isolate has significantly higher replication efficacy than the SD1291 isolate at 72–96 hpi (*p* < 0.05) (Figure 3).

### 3.3. Genomic Comparison

**Figure 4:**
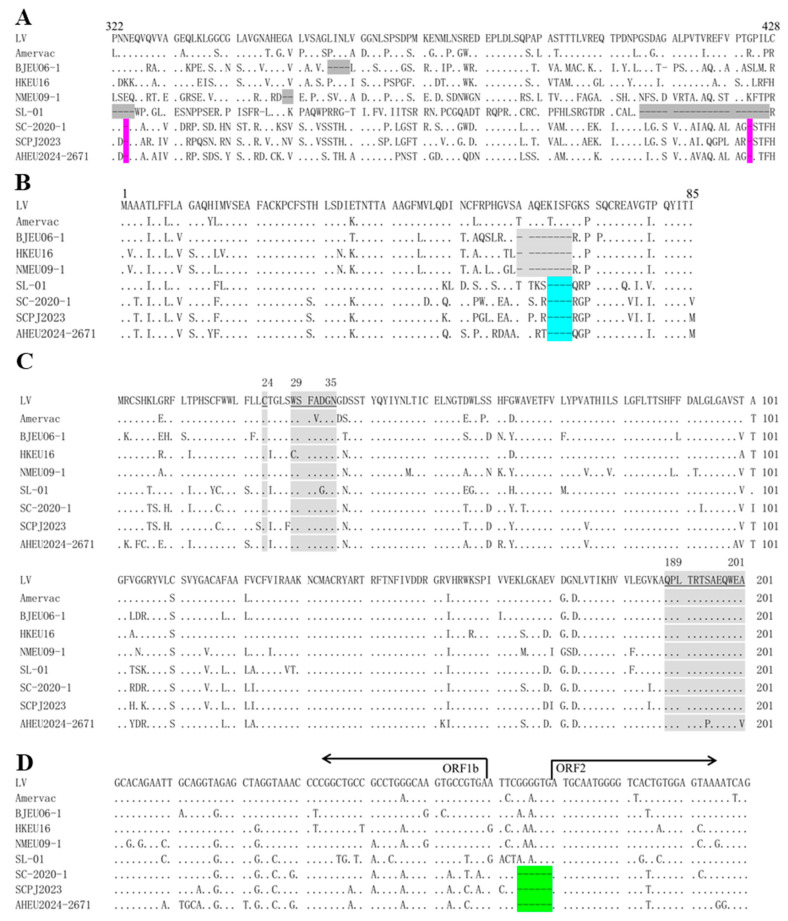
Multiple sequence alignments of representative PRRSV-1 strains. (**A**) The AHEU2024-2671 isolate has 2 single aa deletions at 324 and 423 positions (in pink) of nsp2. The grey highlights are deletions in nsp2 of other PRRSV-1 isolates. (**B**) The AHEU2024-2671 isolate has 4 aa deletions (in turquoise) at positions 64–67 in the GP3-GP4 overlap region. The grey highlights are corresponding deletions in other PRRSV-1 isolates. (**C**) The AHEU2024-2671 isolate has 31 aa substitutions compared with the LV strain, including two mutations within the 189-201 B-cell epitope. The grey highlights are the B-cell epitope sites in GP5. (**D**) The AHEU2024-2671-like isolates have a unique 4 nt deletion (green) between theORF1b gene and the ORF2 gene.

To determine the genomic feature of the new PRRSV-1 isolate, the complete genome of AHEU2024-2671 was identified. The AHEU2024-2671 genome is 15,074 bp in length excluding the poly (A) tail. The genome sequence of AHEU2024-2671 has been deposited into the GenBank database with the accession number PQ640355. In addition, ORF5 sequencing confirmed that the other two PRRSV-1-positive clinical samples contain nearly identical viruses to AHEU2024-2671 (100% ORF5 identity). As shown in Table 3, the AHEU2024-2671 isolate only shared 86.67% and 84.06% genome similarities with the representative LV strain and the predominant BJEU06-1 isolate in China, respectively. Noticeably, the AHEU2024-2671 isolate had the highest genome homology (90.67%) with the SC2020-1 isolate. Each fragment comparison showed that nsp2TF and GP3 are the most variable nonstructural and structural proteins, respectively. As shown in Figure 4, multiple nsp2 alignment showed that the AHEU2024-2671 isolate had the same single amino acid (aa) deletions at 324 and 423 positions as SC2020-1 and SCPJ2023 isolates (Figure 4A). In addition, a 4-aa deletion was also identified at 64–67 positions within the GP3 and GP4 overlap regions of the AHEU2024-2671, SL-01, SC2020-1 and SCPJ2023 isolates (Figure 4B). Moreover, 31 aa substitutions were observed in GP5 of the AHEU2024-2671 isolate when compared with the LV strain, including 2 aa mutations (S_194_P and A_201_V) within a B-cell epitope (189–201 aa) (Figure 4C). Remarkably, here, we also identified for the first time that there is a 6 nt deletion between ORF1b and ORF2 genes in the genomes of AHEU2024-2671, SC2020-1 and SCPJ2023 isolates (Figure 4D).

### 3.4. Phylogenetic Analysis

**Figure 5:**
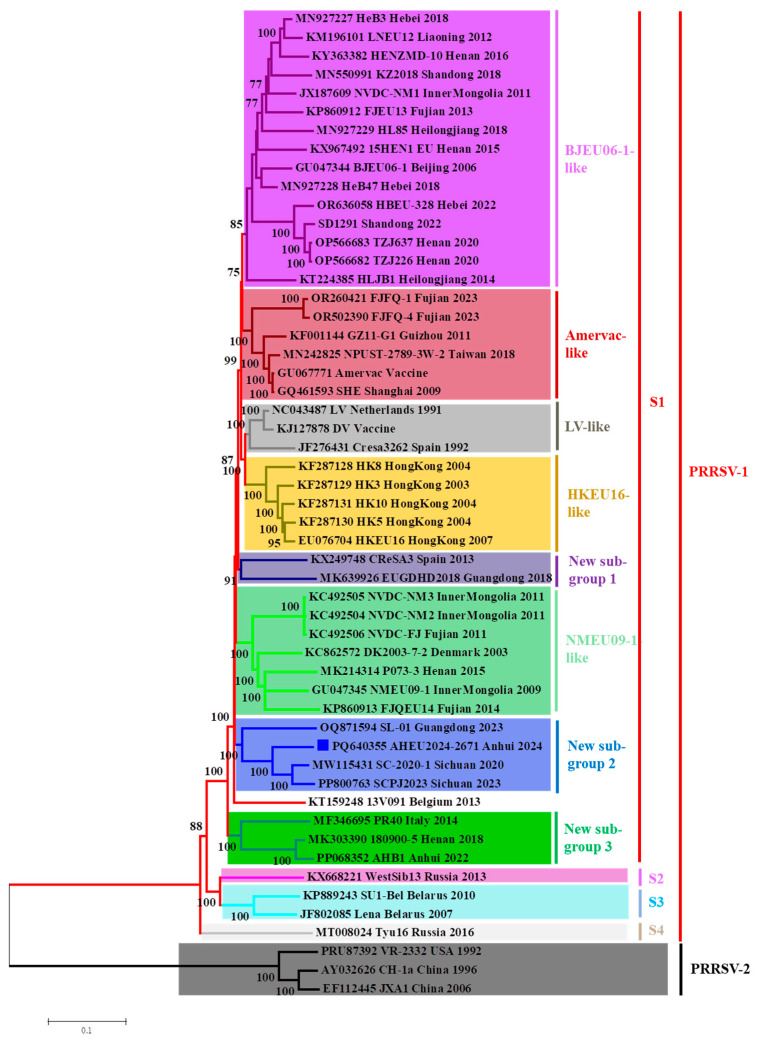
Genome-based phylogenetic tree for Chinese PRRSV-1 isolates. The phylogenetic tree was constructed based on 50 PRRSV-1 and 3 PRRSV-2 genomes. The AHEU2024-2671 isolate was grouped within a new subgroup.

To evaluate the evolutionary relationships between the AHEU2024-2671 isolate and other PRRSV-1 isolates, a genome-based phylogenetic tree was constructed (Figure 5). In addition to the previously described five subgroups, including LV-like, Amervac-like BJEU06-1-like, HKEU16-like and NMEU09-1-like isolates, recent Chinese PRRSV-1 isolates were also clustered within three new subgroups. The AHEU2024-2671 isolate was grouped with SC2020-1, SL-01 and SCPJ2023 isolates, forming a new subgroup 2 within subtype 1 of PRRSV-1 (Figure 5).

### 3.5. Recombination Detection

To detect the role of recombination in the generation of the AHEU2024-2671 isolate, potential cross-over events were detected by RDP4 and SimPlot methods using the 53 multiple aligned PRRSV genomes. No cross-over event was detected in the AHEU2024-2671 genome using both methods.

## 4. Discussion

Even though PRRSV-2 isolates are still predominant in China, a recent review showed that PRRSV-1 has been spread to at least 23 regions in China [32]. More importantly, novel PRRSV-1 isolates with high genetic and pathogenic diversity have kept emerging in Chinese swine herds in recent years [24,30,31,48,49,50]. Therefore, it is extremely important to monitor the prevalence and evolution of PRRSV-1 in China. In this study, we routinely detected PRRSV-1 from February 2022 to May 2024. Within 1521 clinical samples collected from 12 regions in China, only 3 PRRSV-1-positive samples were detected from severely diseased piglets. No other common porcine viruses were detected in these PRRSV-1-positive samples. The new PRRSV-1 strain (AHEU2024-2671) could be isolated in PAMs but not in Marc-145 cells. Genome sequencing and comparison showed that the AHEU2024-2671 isolate showed the highest genome similarity (90.67%) with the SC2020-1 isolate. In addition, aa deletions were identified in nsp2 and the GP3-GP4 overlap region. Noticeably, a novel deletion was first identified between ORF1b and ORF2 genes in this study. Furthermore, phylogenetic analysis indicated that the AHEU2024-2671 isolate is a novel isolate belonging to a new subgroup within subtype 1 of PRRSV-1 isolates. However, no recombination event was detected in the AHEU2024-2671 genome.

Our previously identified PRRSV-1, BJEU06-1 and NMEU09-1 strains were also isolated in PAMs but could not infect Marc-145 cells [22]. In addition, the HLJB1 strain recombined from the BJEU06-1-like isolate and the Amervac vaccine-like isolate also could not replicate in Marc-145 cells [41,51]. Similarly, our AHEU2024-2671 isolate could only be isolated in PAMs but not in Marc-145 cells. However, the Amervac-like GZ11-G1 isolate could be isolated within Marc-145 cells [29]. Similar results were obtained from a previous study by comparing PRRSV isolation in ZMAC and Marc-145 cells [43]. Noticeably, a previous study showed that the 88/94/95 amino acid substitutions in GP2a play a critical role in determining PRRSV-1 adaptation to Marc-145 cells [52]. However, they only evaluated the influences of the triple substitutions in two PRRSV-1 isolates (13V091 and IVI-1173) that are already able to infect Marc-145 cells. Whether these substitutions are sufficient or essential factors for AHEU2024-2671 adaptation to Marc-145 cells deserves further investigation.

PRRSV-1 was first isolated in the Netherlands more than 30 years ago and is still circulating in many European countries such as the Netherlands, Italy and Hungary [9,17,42]. In China, previous epidemiological investigations showed that PRRSV-1-positive rates could range from 0.26% to 32% in different regions, while the majority of studies indicated that the PRRSV-1-positive rate was generally less than 3% [24,48,50,53,54,55,56,57]. For instance, the PRRSV-1-positive rate might reach up to 32% during an outbreak on Taiwan pig farms [48]. In addition, PRRSV-1 was detected in 186 out of 750 samples (24.8%) collected from 50 pig farms in Guangdong province in 2016 [57]. However, an investigation by OIE reference laboratory showed that the percentages of PRRSV-1-positive samples were 1.4% (53/3823) and 2.5% (119/4764) on breeding pig farms from 2018 to 2019 [30]. Our previous studies using 712 clinical samples and 257 clinical samples showed that PRRSV-1-positive rates were 0.28% and 0.39%, respectively [24,54]. In this study, the PRRSV-1-positive rate was only 0.197% during the detection of 1521 clinical samples, indicating that PRRSV-1 is sporadically spread in China.

Nsp2 is the most variable nonstructural protein. Insertions and deletions are commonly detected in the nsp2 of PRRSV-1 isolates [49]. In this study, we also identified two single aa deletions within nsp2 of the AHEU2024-2671 isolate. The nsp2 deletions are identical to previously isolated SC2020-1 and SCPJ2023 strains [31]. Similarly, the same deletion was also detected in the GP3-GP4 overlap region between the AHEU2024-2671 isolate and three previous isolates (SL-01, SC2020-1 and SCPJ2023 strains). Remarkably, positions 48–76 of the GP3-GP4 overlap region was identified as a B-cell epitope site (ES12) previously [58]. The deletion in the ES12 might disturb humoral immune responses by causing conformational changes [59]. Moreover, a novel deletion was first identified between ORF1b and ORF2 genes among AHEU2024-2671, SC2020-1 and SCPJ2023 strains in this study. The exact influences of these deletions on the novel AHEU2024-2671-like isolates deserve further investigation.

Until 2017, Chinese PRRSV-1 isolates were clustered within five subgroups (LV-like, HKEU16-like, BJEU06-1-like, NMEU09-1-like and Amervac-like) [41]. Within this, BJEU06-1-like isolates are predominant in Chinese swine herds [32]. However, more and more novel PRRSV-1 isolates have been detected in China in recent years. Several novel PRRSV-1 genomes have been submitted to GenBank. However, the genomic and pathogenic characteristics of these novel PRRSV-1 isolates were rarely described. In this study, we showed that novel Chinese PRRSV-1 isolates might be divided into three new subgroups within subtype 1 of PRRSV-1. The AHEU2024-2671 isolate was grouped with SL-01, SC2020-1 and SCPJ2023 isolates, forming the new subgroup 2. Our results are consistent with previous studies that novel PRRSV-1 keeps emerging in Chinese swine herds, which deserves more attention.

The novel AHEU2024-2671 isolate has higher in vitro replication efficacy than the BJEU06-1-like SD1291 isolate. The SD1291 isolate has been determined to be a moderately pathogenic strain in piglets [24]. More importantly, the AHEU2024-2671 strain was isolated from severely diseased piglets that tested negative for other common porcine viruses. In addition, our AHEU2024-2671 isolate shared the highest genomic similarity with the SC2020-1 isolate, which was closely associated with a 15% abortion rate in sows from Sichuan province [31]. Whether the AHEU2024-2671 isolate had increased pathogenicity and was responsible for clinically severe diseases requires further investigation.

## 5. Conclusions

This study isolated a novel PRRSV-1 isolate from severely diseased piglets in 2024. Complete genome characterization identified novel deletions in the AHEU2024-2671 genome for the first time. In addition, a genome-based phylogenetic tree showed that the AHEU2024-2671 isolate is a novel PRRSV-1 isolate belonging to a new subgroup within subtype 1 of PRRSV-1 isolates. The pathogenicity of the AHEU2024-2671 isolate should be determined to evaluate whether it is responsible for severe clinical diseases.

## Figures and Tables

**Table 1:** PRRSV-1 detection in 1521 samples collected from February 2022 to May 2024.

Region (City, Province)	Sample No.	PRRSV1-Positive No.	Percentage (%)
Fuyang, Anhui	3	3	100
Zhumadian, Henan	329	0	0
Qingdao, Shandong	213	0	0
Jinchang, Gansu	108	0	0
Neijiang, Sichuan	87	0	0
Yangzhou, Jiangsu	77	0	0
Shuangyashan, Heilongjiang	62	0	0
Hangzhou, Zhejiang	40	0	0
Chaozhou, Guangdong	567	0	0
Beijing	24	0	0
Xianyang, Shanxi	4	0	0
Shihezi, Xinjiang	7	0	0
Total	1521	3	0.197

**Table 2:** Primers used for PRRSV-1 genome amplification.

Primers	Sequence (5′→3′)	Length (bp)	Region *
PRRSV1-1F	ATGATGTGTAGGGTATTCCCC	21	1–1375
PRRSV1-1R	CCATACCACTTGTGTGTCCC	20	
PRRSV1-2F	TCGATCCTGATGGTCCCAT	19	1195–2588
PRRSV1-2R	GTTGTCGGGTGTTTGCTCT	19	
PRRSV1-3F	AAGGTCCTGATGAACAAGCAC	21	2335–3680
PRRSV1-3R	GCTCTTTTGCTCTGTCGC	18	
PRRSV1-4F	TTGGAGAGGTCTCATGCTTTC	21	3491–6279
PRRSV1-4R	CACTGTTGGTCATAGCAAGG	20	
PRRSV1-5F	GCCTCTCGACTGTTCAACT	19	5908–7371
PRRSV1-5R	GGAGTTGACTAATGATGCGC	20	
PRRSV1-6F	GTTGGCACTGTTGTGATCG	19	7176–8533
PRRSV1-6R	GAATTTGTTTTTCCCCAAGGC	21	
PRRSV1-7F	CAAGGAGAATTGGCAAACTG	20	8344–9770
PRRSV1-7R	GCCCCACTATAAACTTGCTG	20	
PRRSV1-8F	ATAACAAAACAACGGCCCT	19	9573–10,975
PRRSV1-8R	TATGCGTCCTGTTGAAACG	19	
PRRSV1-9F	CACCAGAATAATCGGGCG	18	10,766–12,106
PRRSV1-9R	ACCATTTCATCAATTAGGTGGG	22	
PRRSV1-10F	CGCCTTCACTGAGTTCCTT	19	11,830–13,284
PRRSV1-10R	GATGACTTTGAAGCCTTTCTCG	22	
PRRSV1-11F	CGGCCATTCTTTTCCTCC	18	12,941–14,022
PRRSV1-11R	CTTCGAGGACGACATGTTTG	20	
PRRSV1-12F	CTGGGTTTTCTCACAACAAGC	21	13,710–15,074
PRRSV1-12R	AATTTCGGTCACATGGTTC	19	

* The region indicates the location of each amplicon within the genome of AHEU2024-2671 isolate. The primers were modified from previous studies [22,24,41].

**Table 3:** Detailed comparisons of AHEU2024-2671 isolate to representative PRRSV-1 strains.

Region	Length *	Similarity to AHEU2024-2671 (%)					
		LV	Amervac	BJEU06-1	HKEU06	NMEU09-1	SC2020-1
Nucleotides (nt)							
5′UTR	221	92.34	91.89	91.44	91.44	90.54	91.44
ORF1a	7185	85.18	83.41	81.82	81.88	79.82	89.23
ORF1b	4392	88.39	87.30	86.13	86.20	84.45	92.94
ORFs 2–7	3177	87.21	86.52	85.55	86.37	85.99	90.78
3′UTR	114	94.74	92.11	94.74	92.98	94.74	96.49
Complete	15,074	86.67	85.34	84.06	84.24	82.68	90.67
Proteins (aa)							
Nsp1α	180	92.22	90.56	89.44	87.78	90.56	90.00
Nsp1β	205	81.95	80.00	80.00	79.02	75.61	82.44
Nsp2N	729	76.06	73.87	69.04	69.90	65.53	82.58
Nsp2TF	898	64.27	62.68	58.45	73.89	70.67	84.86
Nsp2	1076	82.00	80.24	76.60	77.46	73.84	86.73
Nsp3	230	92.61	92.61	90.43	92.17	91.74	94.78
Nsp4	203	91.13	92.12	87.68	89.66	84.24	94.09
Nsp5	170	93.53	91.76	92.94	93.53	90.00	95.29
Nsp6	16	93.75	93.75	93.75	100.00	93.75	100.00
Nsp7α	149	95.97	95.30	95.97	94.63	96.64	97.32
Nsp7β	108	92.59	89.81	92.59	92.59	88.89	91.67
Nsp8	45	95.56	93.33	93.33	93.33	95.56	97.78
Nsp9	685	95.62	95.33	95.18	95.18	94.74	96.06
Nsp10	442	93.44	92.99	94.57	91.18	92.31	96.38
Nsp11	224	96.88	96.88	96.88	94.64	94.64	96.88
Nsp12	152	93.42	94.08	92.76	93.42	90.13	94.74
GP2a	249	89.96	87.55	88.35	87.15	87.15	94.38
E	70	94.29	94.29	94.29	90.00	94.29	98.57
GP3	261	78.60	78.60	77.12	78.87	80.07	84.87
GP4	179	83.06	84.15	80.33	83.06	83.06	84.70
GP5	201	84.58	86.57	88.56	88.56	85.57	88.06
GP5a	43	95.35	93.02	88.37	93.02	95.35	90.70
M	173	91.91	91.91	89.60	93.64	91.33	91.33
N	128	92.97	92.19	85.94	89.06	88.28	90.63

* The length of each fragment in the AHEU2024-2671 genome. The cleavage products were determined based on previous studies [1,46,47].

## Data Availability

The genome sequence of the PRRSV-1 AHEU2024-2671 isolate has been deposited into the GenBank database with the accession number PQ640355.

## Supplementary Materials

The following supporting information can be downloaded at https://www.mdpi.com/article/10.3390/vetsci12010061/s1. Table S1: Primers and probes used for the detection of porcine viruses.
