# Supplementary material for: Isolation and Genomic Characterization of a Novel Porcine Reproductive and Respiratory Syndrome Virus 1 from Severely Diseased Piglets in China in 2024
Source: Vet Sci. 2025 Jan 15;12(1):61. doi: 10.3390/vetsci12010061 (PMC11769002; doi:10.3390/vetsci12010061)
Supplement: Supplementary file 1 [file vetsci-12-00061-s001.zip › vetsci-3367957-supplementary.pdf]

Table S1. Primers and probes used for the detections of porcine viruses

Primers	Sequence (5'→3')	Length (bp)
PRRSV1-UF	CAGATGCAGATTGTGTTGCCT	20
PRRSV1-UR	ATGGAGACCTGCAGCACTTTC	21
PRRSV-UP	FAM-TCTGGCCCCTGCCCA-MGB	15
PRRSV2-UF	TTGTGCTTGCTAGGCCGC	18
PRRSV2-UR	ACGACAAATGCGTGGTTATCA	21
PRRSV-UP	FAM-TCTGGCCCCTGCCCA-MGB	15
CSFV-WF	AACTGGGCTAGCCATGCCCACAGT	24
CSFV-WR	TGTACTCAGGACTTAGACCACCCA	24
CSFV-WP	FAM-CGCCACTACGGCTAGT-MGB	16
PEDV-F	GGACACATTCTTGGTGGTCT	20
PEDV-R	GCCATAAAGTTTCTGTTTAGACTAA	25
PEDV-P	FAM-CTGCTTTAGGAACAAAT-MGB	35
TGEV-F	AATATGGAAGACCTCAATTCAGCTG	25
TGEV-R	CGCACTACTTGCTTTCAACTTCTT	24
TGEV-P	ROX-TCACGTTCACACACAAATACCACTTGCCAA-BHQ2	30
PDCoV-F	ACTGAGAAGACGGGTATGGCT	21
PDCoV-R	TGGTATAATCAACCTTCTAGCACTGT	26
PDCoV-P	TAMRA-CCACAGGATATGGTGATAGTGTCACCAAGTGA-BHQ2	31
ASFV-F	TCTCTTGCTCTGGATACGTTAATATGAC	28
ASFV-R	CGGGTGCGATGATGATTACC	20
ASFV-P	FAM-ACTGGGTTGGTATTCCT-MGB	17
PRV-gE-F	AGCTTCCACTCGCAGCTCTTCT	22
PRV-gE-R	TGAAGTTCTCGCGCGAGTC	19
PRV-gE-P	VIC-ACACGTTCGACCTGATG-MGB	17
PCV2-F1	CAGGGCCAGAATTCAACCTTAA	22
PCV2-R1	TTTCTTCCCCCAGGAGGG	18
PCV2-P	ROX-TTAAGTGGGGGGTCTTTAAGATTAAATTCTCTGAATTGT-BHQ2	39
PCV3-F1	CGGTGGGGTCATATGTGTTGA	21
PCV3-R1	AAACACAGCCGTTACTTCACCC	22
PCV3-P	HEX-ATATGTGTTGAGCCATGGGGTGGGTCT-BHQ1	27
