# Supplementary figures and images for: Isolation and Genomic Characterization of a Novel Porcine Reproductive and Respiratory Syndrome Virus 1 from Severely Diseased Piglets in China in 2024
Source: Vet Sci. 2025 Jan 15;12(1):61. doi: 10.3390/vetsci12010061 (PMC11769002; doi:10.3390/vetsci12010061)

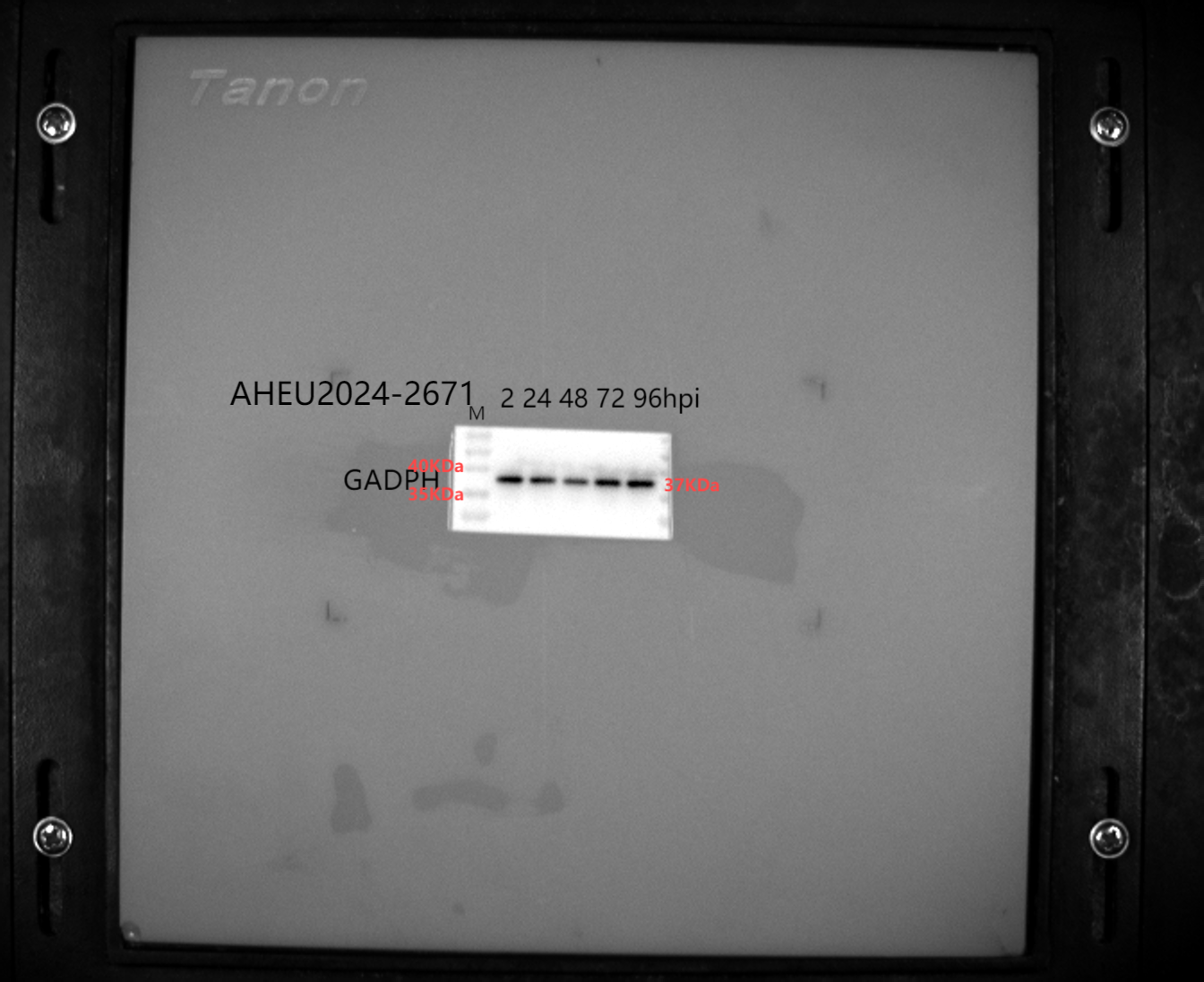

Supplement: Supplementary file 1 [file vetsci-12-00061-s001.zip › GAPDH AH 2-96hpi 420MS 2024.09.04.png]

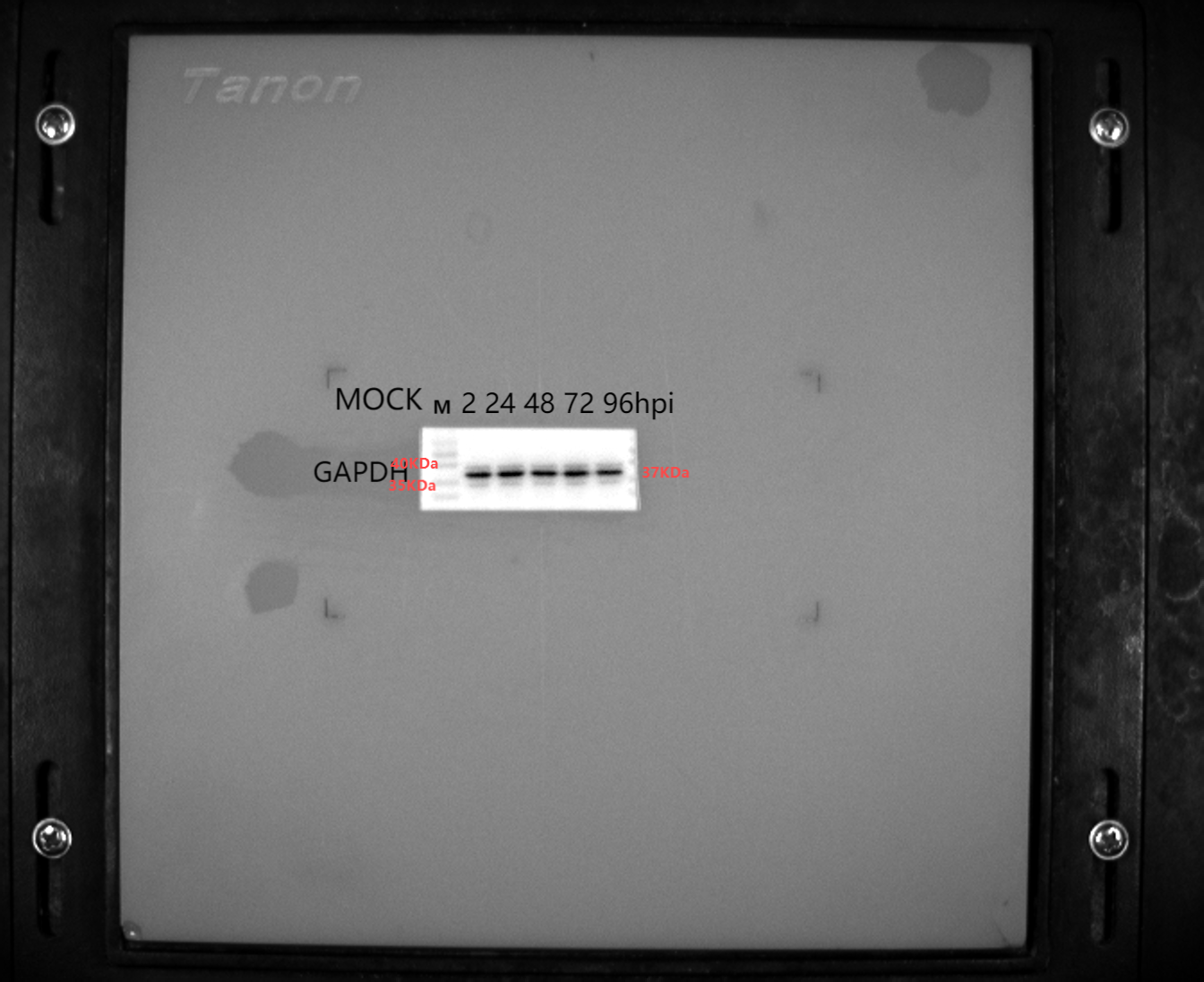

Supplement: Supplementary file 1 [file vetsci-12-00061-s001.zip › GAPDH Mock 2-96hpi 420MS 2024.09.04.png]

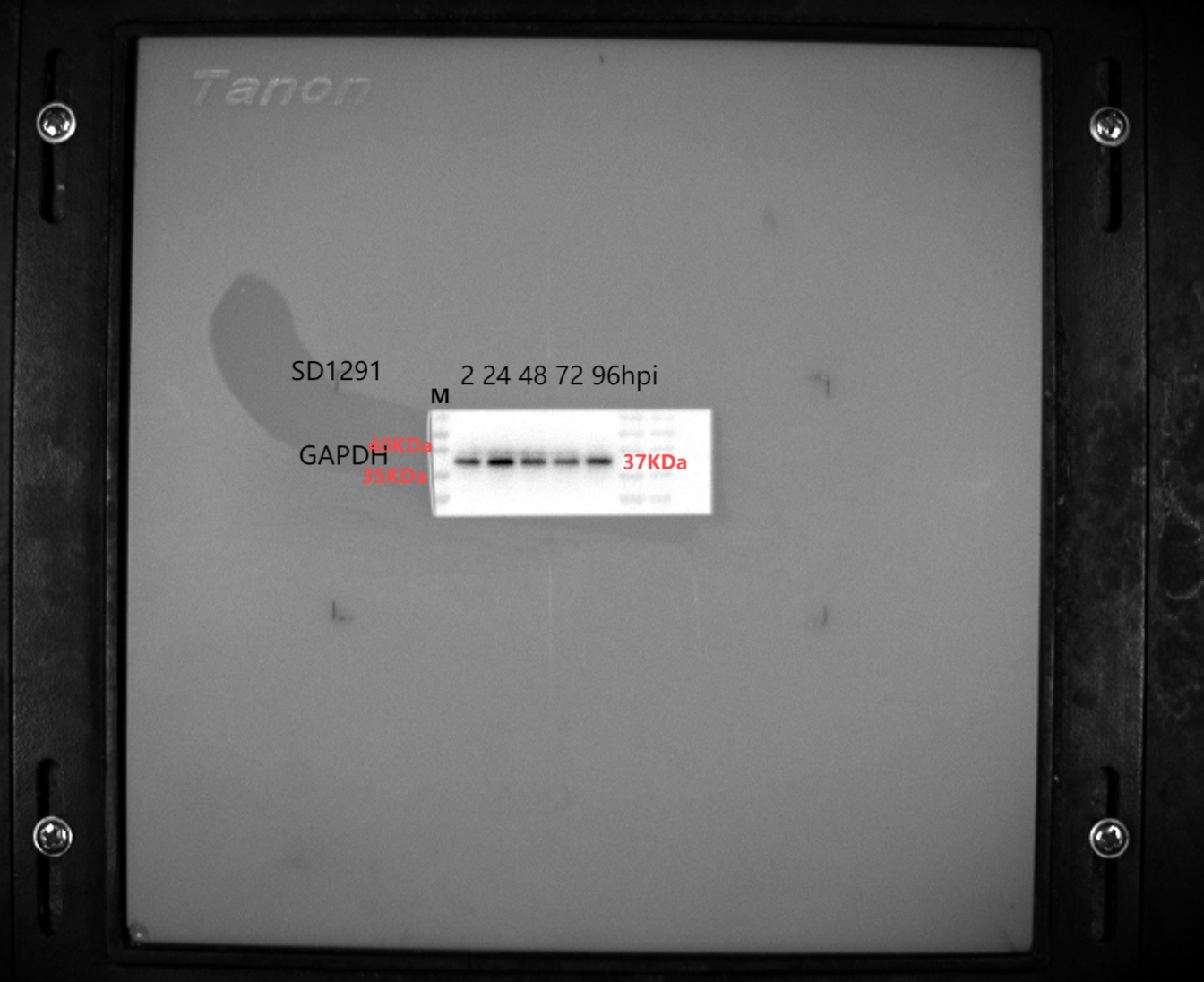

Supplement: Supplementary file 1 [file vetsci-12-00061-s001.zip › GAPDH SD 2-96hpi 420MS 2024.09.04.png]

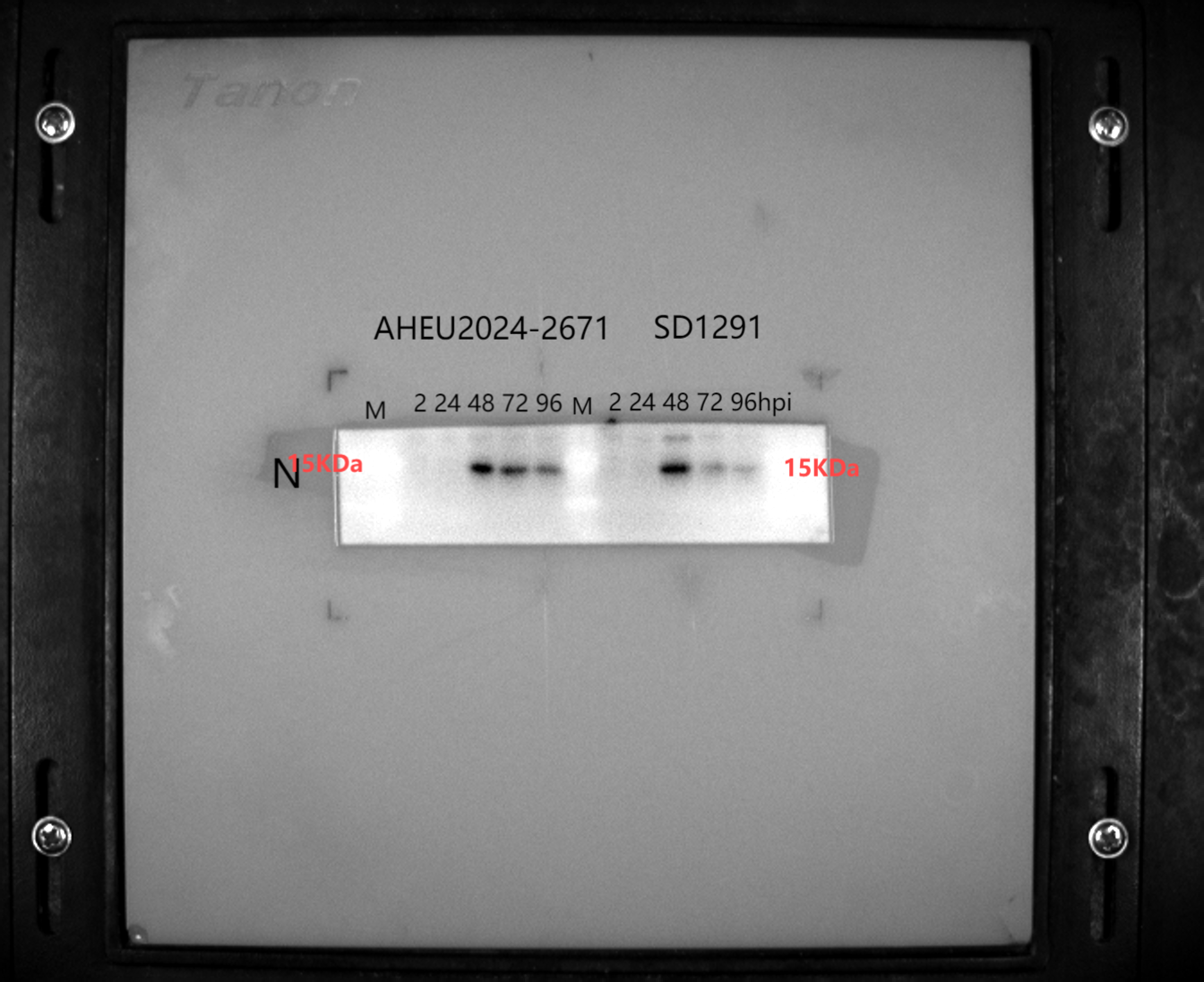

Supplement: Supplementary file 1 [file vetsci-12-00061-s001.zip › N protein AH SD 2-96hpi 25S 2024.09.04.png]

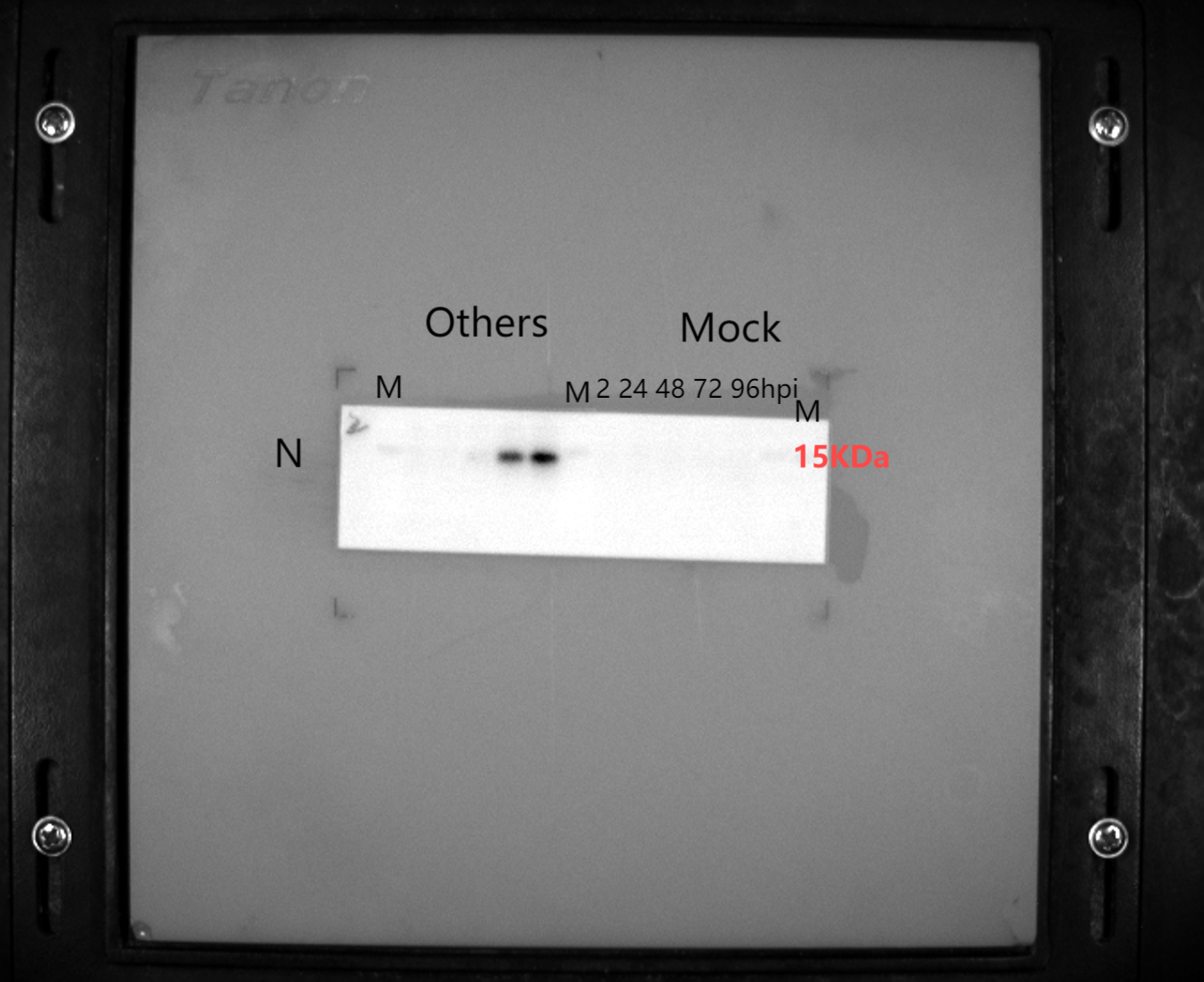

Supplement: Supplementary file 1 [file vetsci-12-00061-s001.zip › N protein Mock 2-96hpi 4S 2024.09.04.png]
